# Candidate gene analysis in pathogenesis of surgically and non-surgically treated necrotizing enterocolitis in preterm infants

**DOI:** 10.1007/s11010-017-3135-5

**Published:** 2017-08-02

**Authors:** Dawid Szpecht, Natalia Neumann-Klimasińska, Michał Błaszczyński, Agnieszka Seremak-Mrozikiewicz, Grażyna Kurzawińska, Dorothy Cygan, Marta Szymankiewicz, Krzysztof Drews, Janusz Gadzinowski

**Affiliations:** 1 Chair and Department of Neonatology, Poznan University of Medical Sciences, Poznan, Poland; 2 Department of Pediatric Surgery, Traumatology and Urology, Poznan University of Medical Sciences, Poznan, Poland; 3 Department of Perinatology and Women’s Diseases, Poznan University of Medical Sciences, Poznan, Poland; 4 Department of Pharmacology and Phytochemistry, Institute of Natural Fibers and Plants, Poznan, Poland; 5 Poznan University of Medical Sciences, Poznan, Poland

**Keywords:** Gene, Polymorphism, Necrotizing enterocolitis, Preterm newborn

## Abstract

Necrotizing enterocolitis (NEC) is one of the most severe and unpredictable complications of prematurity. There are two possible mechanisms involved in the pathogenesis of NEC: individual inflammatory response and impaired blood flow in mesenteric vessels with secondary ischemia of the intestine. The aim of this study was to evaluate the possible relationship between polymorphisms: Il-1β *3953C*>*T*, Il-6 −*174G*>*C* and −*596G*>*A*, TNFα −*308G*>*A*, and 86 bp variable number tandem repeat polymorphism of interleukin-1 receptor antagonist (*Il-1RN VNTR 86 bp*) and three polymorphisms that may participate in arteries tension regulation and in consequence in intestine blood flow impairment: eNOS (*894G*>*T* and −*786T*>*C*) and END-1 (*5665G*>*T*) and NEC in 100 infants born from singleton pregnancy, before 32 + 0 weeks of gestation, exposed to antenatal steroids therapy, and without congenital abnormalities. In study population, 22 (22%) newborns developed NEC. Surgery-requiring NEC was present in 7 children. Statistical analysis showed 20-fold higher prevalence of NEC in infants with the genotype *TT* [OR 20 (3.71–208.7); *p* = 0.0004] of eNOS *894G*>*T* gene polymorphism. There was a higher prevalence of allele *C* carriers of eNOS *786T*>*C* in patients with surgery-requiring NEC [OR 4.881 (1.33–21.99); *p* = 0.013]. Our investigation did not confirm any significant prevalence for NEC development in another studied genotypes/alleles. This study confirms the significant role of polymorphisms that play role in intestine blood flow. Identifying gene variants that increase the risk for NEC development may be useful in screening infants with inherent vulnerability and creating strategies for individualized care.

**Electronic supplementary material:**

The online version of this article (doi:10.1007/s11010-017-3135-5) contains supplementary material, which is available to authorized users.

## Background

Necrotizing enterocolitis (NEC) is one of the most severe and unpredictable complications of prematurity. 7% of neonates born with very low birth weight (VLBW) will develop NEC [1]. Despite the efforts, up to 1/3 of the cases will be fatal [2] and the rest remain threatened by chronic gastrointestinal complications and poor neurodevelopmental outcome [3]. Several risk factors for developing NEC have been reported, although the exact pathogenesis of this disease remains unclear. Onset of clinical symptoms might be abrupt and the severity of the disease may lead to surgical intervention. Thereby the morbidity and mortality tends to be higher in patients requiring laparotomy [2, 4].

According to the present consensus, NEC develops in the premature intestine in the setting of improper bacterial colonization, often after enteral feedings with formula instead of breast milk. A baseline increased reactivity of the premature intestinal mucosa to microbial ligands leads to increased and flawed inflammatory response [5, 6]. The proof of this theory seems to be the fact that plasma concentrations and tissue expression of the proinflammatory cytokines interleukin 1β (Il-1β) and interleukin 6 (Il-6), as well as tumor necrosis factor alpha (TNF-α) [7], are elevated in neonates with NEC [8, 9].

Another somewhat controversial hypothesis involves hypoxia/ischemia injury [10]. Nitric oxide (NO), a product of endothelial (eNOS) and inducible NO (iNOS) synthases [11], has a crucial role in gut physiology. Not only is NO responsible for the tension of vascular wall, but it also promotes damage to the intestinal barrier by inducing apoptosis of enterocytes and inhibiting their regeneration [12]. NO might be produced through the activity of commensal intestinal bacteria [13]. Breast milk, with its rich levels of nitrate, may have a protective role against NEC due to its vasodilatory effect [14]. In state of chronic inflammation and oxidative stress, which characterizes late NEC, mesenteric production of NO decreases and contributes to further vasoconstriction and intestinal ischemia [15]. Nitric oxide synthase is crucial for intestinal microcirculation and therefore, it may play a key role in the pathogenesis of NEC [16]. On the contrary, endothelin 1 (END-1) is the strongest known vasoconstrictor and plays a major role in maintaining hemodynamic homeostasis by changing the distribution of blood in the system [17].

We propose that the recognition of Single Nucleotide Polymorphisms (SNP) for inflammatory response markers: Il-1β, Il-6, TNF-α, Il-1RN, as well as vasoactive agents eNOS and END-1, might add insight to the pathogenesis of NEC. SNPs, single base pair changes, occur naturally in the human genome and can alter the translation of related proteins, influencing various processes. Since studies on animal models have proposed that modulating the inflammatory cytokine cascade could be profitable for treating preterm neonates with NEC [18, 19], undoubtedly better understanding of the diversity of NEC patients’ biology could lead to enhancement of individualized treatment options.

## Materials and methods

### Population

**Fig. 1:**
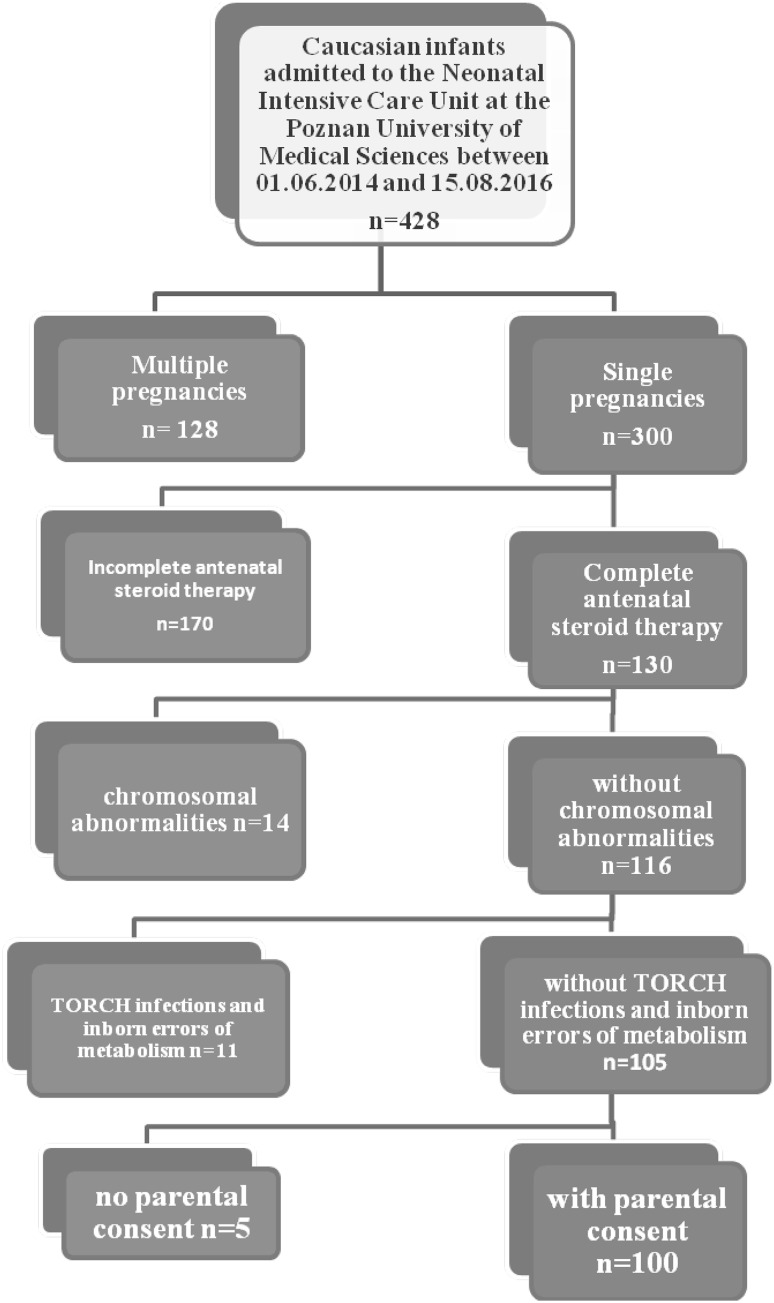
Inclusion and exclusion criteria for study

In order to guarantee a homogenous group of patients, we used the following inclusion criteria: Caucasian origin, neonates born between 24 + 0 and 32 + 0 weeks of gestation, single pregnancy neonates, pregnancies without death of one of the fetuses, and newborns with antenatal steroid therapy (AST). Newborns with chromosomal abnormalities, TORCH infections (*toxoplasmosis*, *other*, *rubella*, *cytomegalovirus*, *herpes*), and inborn errors of metabolism were excluded from this study—Fig. 1. We enrolled 100 (23.3%) out of the 428 infants admitted to the Neonatal Intensive Care Unit at the Poznan University of Medical Sciences between June 1st, 2014 and August 15th, 2016.

### Additional data

We collected the following information: gender, gestational age (GA; weeks), birth weight (BW, g), birth weight under 3rd percentile, the type of delivery (vaginal vs. cesarean section), birth asphyxia (defined as APGAR score <6 at 10 min and pH <7.0 or blood base excess (BE) <−12 mmol/l in cord blood), intrauterine infection (defined as positive blood culture accompanied by clinical symptoms or inborn pneumonia diagnosed in the first 48 h after birth), surfactant replacement therapy (indications for the treatment based on European guidelines [20]), ventilatory support (conventional vs. non-invasive), intraventricular hemorrhage (IVH, definition based on Papile classification [21]), and the presence of bronchopulmonary dysplasia (definition by the National Institute of Child Health and Human Development [22]). Our patients were fed according to a protocol proposed by ESPHGAN [23].

### Diagnosis of NEC

The diagnosis was based on Modified Bell’s staging criteria for NEC [24]. Auxiliary examination in the diagnosis of NEC was used as an abdomen ultrasound (5–8 and 13 MHz transducer, Prosound α7 Premier, Aloka) and X-ray of the abdomen. Abdomen ultrasounds were performed by a single trained neonatologist.

### Surgical treatment

Every patient developing the symptoms of mild, moderate, or severe forms of NEC was consulted by the pediatric surgeon. Qualification for surgical treatment was based on past history, symptoms, and results of laboratory and imaging studies. Surgical procedures included laparotomy, resection of necrotic intestine, and intestinal anastomosis with decompressing temporary enterostomy using T-tube (TTES) [25]. In case of multilocal or extensive necrosis found during laparotomy, the proximal enterostomy with Hartmann procedure was performed. In critically ill and unstable premature children, the peritoneal drainage was used as a primary and definitive procedure or as the first step in surgical treatment.

### Ethical considerations

Informed consent was obtained from the parents of all infants enrolled in the study.

The study followed the tenets of the Declaration of Helsinki and was approved by the Bioethics Committee of Poznan University of Medical Sciences (66/14 and 799/16).

### Laboratory method

Our selection of candidate genes is based on two possible mechanisms involved in the pathogenesis of NEC: individual inflammatory response and impaired blood flow in mesenteric vessels with secondary ischemia of the intestine. Accordingly, we analyzed the following polymorphisms: Il-1β *3953C*>*T*, Il-6 −*174G*>*C* and −*596G*>*A*, TNF-α −*308G*>*A*, and 86 bp variable number tandem repeat polymorphism of interleukin-1 receptor antagonist (*Il-1RN VNTR 86 bp*) and three polymorphisms that may participate in arterial tension regulation and in consequence in intestinal blood flow impairment: eNOS (*894G*>*T* and −*786T*>*C*) and END-1 (*5665G>T*).

A blood sample (0.5 ml) was taken directly post-delivery and banked. Genomic DNA was extracted from blood leukocytes using QIAamp DNA Blood Mini Kit (QIAGEN inc; Germany) according to the manufacturer’s recommendations. Genotyping was performed using polymerase chain reaction–restriction fragment length polymorphism (PCR–RFLP) procedures. Primer sequences and conditions for PCR–RFLP analyses and restriction fragment length are presented in Table 1. Products were analyzed by electrophoresis on 2% agarose gel with Midori Green Advanced DNA Stain (Nippon Genetics, Europe GmbH).

**Table 1:** Description of the studied polymorphisms

Gene symbol	Polymorphism	Sequence of primers	Restriction enzyme	Products
Il-1β	*+3953C>T* *(rs1143634)*	F 5′-gTTgTC ATC Aga CTT TgA CC-3′ R 5′-TTC AgT TCA TAT ggA CCA gA-3′	*TaqI*	*CC* 137, 114 bp *CT* 251, 137, 114 bp *TT* 251 bp
Il-1RN	*86 bp VNTR* *(rs2234663)*	F 5′-CTC AgC AAC ACT CCT AT-3′ R 5′-TCC Tgg TCT gCAggT AA-3′		IL1RN*0 154 bp IL1RN*1 410 bp IL1RN*2 240 bp IL1RN*3 500 bp IL1RN*4 325 bp IL1RN*5 595 bp
Il-6	*−174G>C* *(rs1800795)*	F 5′-ACA TgC CAA gTgCTgAgT CA-3′ R 5′-AAT CTT TgTTggAgggTg Ag-3′	*LweI*	*GG* 114, 100 bp *GC* 214, 114, 100 bp *CC* 214 bp
Il-6	*−596G>A* *(rs1800797)*	F 5′-ggAgTC ACA CAC TCC ACC Tg-3′ R 5′-AAgCAg AAC CAC TCT TCC TTT ACT T-3′	*BseGI (BtsCI)*	*GG* 420 bp *GA* 420, 354, 66 bp *AA* 354, 66 bp
TNF-α	*−308G>A* *(rs1800629)*	5′-AAA TggAgg CAA Tag gTTTTgAggggCTTg-3′ 5′-TAC CCC TCA CAC TCC CCA TCC TCCCTg ATC-3′	*FaqI (BsmFI)*	*GG* 86, 45 bp *GA* 131, 86, 45 bp *AA* 131 bp
eNOS	*894G*>*T* *(rs1799983)*	F 5′-AAggCAggAgACAgTggATgg A-3′ R 5′-CCC AgT CAA TCC CTT TggTgC TCA-3′	*MboI*	*GG* 248 bp *GT* 248, 158, 90 bp *TT* 158, 90 bp
eNOS	*−786T*>*C* *(rs2070744)*	F 5′-CCA CCC TgT CAT TCA gTg AC-3′ R 5′-TCT CTgAgg TCT CgA AAT CA-3′	*PdiI*	*TT* 296 bp *TC* 296, 220, 76 bp *CC* 220, 76 bp
EDN1	*5665G*>*T* *(rs5370)*	F 5′-TCA TgA TCC CAA gCTgAAAgg CTA-3′ R 5′-ACC TTT CTT ggAATg TTT TgA AC-3′	*NheI*	*GG* 203, 25 bp *GT* 228, 203, 25 bp *TT* 228 bp

Il-*1β* interleukin-1β, *Il*-*6* interleukin 6, *TNFα* tumor necrosis factor alpha, *Il1 RN* 86 bp variable number tandem repeat polymorphism of interleukin-1 receptor antagonist, *END-1* endothelin-1, *eNOS* endothelial nitric oxide synthase

### Statistical analysis

The normality of variable distribution was assessed by Shapiro–Wilk test. Accordingly, the results are presented as percentage for categorical variables, or median (range) for non-normally distributed continuous variables. *p* value <0.05 indicates statistical significance. The following tests were used to evaluate the association between NEC and categorical variables: the Fisher‘s exact probability test, the Chi-square test, Fisher Freeman Halton, and Chi-squared test with Yates's correction. Differences in non-normally distributed continuous variables were compared with the U Mann–Whitney test. Logistic regression analysis was used to compute ORs and their 95% confidence intervals (CI) for patients without NEC and with NEC combined with different genotypes and alleles. The odds ratio (OR) and 95% confidence intervals (95% CI) were calculated in surgically and non-surgically treated NEC in different genotype distributions of the studied polymorphisms. Statistical analysis was performed using CytelStudio version 10.0, created January 16, 2013 (CytelStudio Software Corporation, Cambridge, Massachusetts, United States), and Statistica version 10, 2011 (Stat Soft, Inc., Tulsa, Oklahoma, United States).

## Results

The median gestational age of enrolled infants was 28 + 4 (range 24 + 0–32 + 0). The median birth weight was 1124.4 ± 378.7 grams. In our study population, we found that 22 (22%) preterm newborns developed NEC. Surgery-requiring NEC was present in 7 children (7% of total, 31.8% of NEC cases). The primary peritoneal drainage was performed in 4 cases. Laparotomy was done in 6 patients: 3 of them as primary operation and in 3 others secondary to peritoneal drainage. In one patient, three surgical procedures were performed: primary peritoneal drainage (PPD), laparotomy with intestinal resection and TTES formation, and relaparotomy with resection and TTES. Finally, in 7 patients, 11 surgeries were done.

The incidence of NEC was comparable in female (11; 50%) and male (11; 50%) neonates with no significance. The incidence of NEC was significantly higher in children born from 24 + 0 to 28 + 6 weeks of gestation than born from 29 + 0 to 32 + 0 weeks of gestation (72.73 vs. 27.27%; *p* = 0.046). Moreover, we confirmed that the lower the gestational age, the greater the lower birth weight (*p* = 0.013); the higher the incidence of NEC. The disease was more prevalent in children ventilated conventionally (68.18 vs. 21.82%; *p* = 0.031).

Ten of 100 (10%) cases were fatal, including 5 patients treated due to NEC. In our Department, the mortality rate of infants with NEC was 22%. In 2 out of 5 deceased neonates (40%), NEC was surgically treated which provides the surgical NEC mortality rate of 28.5%. Table 2 shows clinical and demographic data of enrolled infants.

**Table 2:** Demographic and clinical characteristic of enrolled infants

	Group without NEC *N* = 78 (%)	Group with NEC *N* = 22 (%)	*p* value
Gender			0.670^a^
Male	43 (55.13)	11 (50.00)	
Female	35 (44.87)	11 (50.00)	
Gestational age (week)			0.046^a^
24–28	38 (48.72)	16 (72.73)	
29–32	40 (51.28)	6 (27.27)	
Birth weight (g)			0.013^a^
< 750	9 (11.54)	5 (22.73)	
750–1000	17 (21.79)	10 (45.45)	
>1000	52 (66.67)	7 (31.82)	
Birth weight < 3rd percentile			0.165^b^
Yes	67 (85.90)	16 (72.73)	
No	11 (14.10)	6 (27.27)	
Apgar score (median and range)			0.334^d^ 0.396^d^
1st minute	6 (1–10)	5 (1–9)	
5th minute	7 (1–10)	7 (5–9)	
Mode of delivery			0.052^b^
Vaginal	28 (35.90)	13 (59.09)	
Cesarean section	50 (64.10)	9 (40.91)	
Asphyxia (ph lower than 7.0 or BE lower than −12)			0.458^c^
Yes	3 (4.00)	0 (0.00)	
No	72 (96.00)	22 (100.0)	
Surfactant therapy			0.068^a^
Yes	36 (46.15)	15 (68.18)	
No	42 (53.85)	7 (31.82)	
Ventilation support			0.031^a^
Non-invasive	45 (57.69)	7 (31.82)	
Conventional	33 (42.31)	15 (68.18)	
IVH II–IV			0.036^b^
Yes	20 (25.64)	11 (50.00)	
No	58 (74.36)	11 (50.00)	
BPD			0.043^b^
Yes	24 (30.77)	12 (54.55)	
No	54 (69.23)	10 (45.45)	
Deaths			0.040^b^
Yes	5 (6.49)	5 (22.73)	
No	72 (93.51)	17 (77.27)	

Results are expressed as absolute number of patients (percentage) and median (interquartile range)

*NEC* necrotizing enterocolitis, *IVH* intraventricular hemorrhage, *BPD  *bronchopulmonary dysplasia

^a^Chi-square test

^b^Fisher Freeman Halton test

^c^Chi-square test with Yate’s correction

^d^Mann–Whitney test

 Statistical analysis showed 20-fold higher prevalence of NEC in infants with the genotype *TT* (OR 20 (3.71–208.7); *p* = 0.0004) of eNOS *894G*>*T* gene polymorphism. There was a higher prevalence of allele *C* carriers of eNOS *786T*>*C* in patients with surgery-requiring NEC (OR 4.881 (1.33–21.99); *p* = 0.013). Our investigation did not confirm any significant prevalence for NEC development in other studied genotypes/alleles such as END-1 *5665G*>*T*; Il-1β *3953C*>*T*, Il-6 −*174G*>*C* and −*596G*>*A*, TNF-α −*308G*>*A;* and *Il*-*1RN* *VNTR 86 bp*. Genotype distribution of the polymorphisms involved in inflammation pathways in infants with/without NEC and with/without surgery-requiring NEC is presented in Table 3. Genotype distribution of eNOS (*894G*>*T* and −*786T*>*C*) and END-1 (*5665G*>*T*) in infants without/with NEC and with/without surgery-requiring NEC is presented in Table 4.

**Table 3:** Genotype distribution of the five polymorphisms involved in inflammation pathway in infants without and with NEC and without/with NEC surgery-requiring

Gene symbol db SNP	Genotypes/allele	Group without NEC *N* (%)	Group with NEC *N* (%)	*p*	OR 95% CI *p*	Group with NEC without surgery *N* (%)	Group with NEC with surgery *N* (%)	*p*	OR 95% CI *p*
Il-1β *+3953C>T* *(rs1143634)*	*CC*	47 (60.26)	15 (68.18)	–	References	57 (61.29)	5 (71.43)	–	References
	*CT*	26 (33.33)	5 (22.73)	0.540	0.603 (0.154–2.016)	29 (31.18)	2 (28.57)	1.000	0.786 (0.071–5.182)
	*TT*	5 (6.41)	2 (9.09)	1.000	1.253 (0.108–8.656)	7 (7.53)	0 (0.00)	1.000	0.000 (0.000–10.83)
	*C*	120 (76.92)	35 (79.55)	–	References	143 (76.88)	12 (85.71)	–	References
	*T*	36 (23.08)	9 (20.45)	0.887	0.857 (0.331–2.042)	43 (23.12)	2 (14.29)	0.702	0.554 (0.058–2.646)
*IL1RN* *86 BP* *VNTR* (*rs2234663*)	1/1	40 (51.28)	10 (45.45)	–	References	47 (50.54)	3 (42.86)	–	References
	1/2	26 (33.33)	7 (31.28)	1.000	1.077 (0.306–3.603)	31 (33.33)	2 (28.57)	1.000	1.011 (0.080–9.358)
	1/3	2 (2.56)	0 (0.00)	1.000	0..000 (0.000–23.05)	2 (2.15)	0 (0.00)	1.000	0.000 (0.000–101.6)
	2/2	9 (11.54)	5 (22.73)	0.379	2.222 (0.470–9.455)	12 (12.90)	2 (28.57)	0.599	2.611 (0.194–25.15)
	2/3	1 (1.28)	0 (0.00)	1.000	0.000 (0.000–159.9)	1 (1.08)	0 (0.00)	1.000	0.000 (0.000–624)
	1	108 (69.23)	27 (61.36)	–	References	127 (68.28)	8 (57.14)	–	References
	2	45 (28.85)	17 (38.64)	0.328	1.511 (0.699–3.198)	56 (30.11)	6 (42.86)	0.503	1.701 (0.462–5.874)
	3	3 (1.92)	0 (0.00)	1.000	0.000 (0.000–10.10)	3 (1.61)	0 (0.00)	1.000	0.000 (0.000–42.83)
Il-6 *−174G>C* (*rs1800795*)	*GG*	14 (17.95)	7 (31.82)	–	References	19 (20.43)	2 (28.57)	–	References
	*GC*	49 (62.82)	12 (54.55)	0.326	0.489 (0.144–1.776)	57 (61.29)	4 (57.14)	0.969	0.667 (0.088–7.963)
	*CC*	15 (19.23)	3 (13.64)	0.414	0.400 (0.057–2.250)	17 (18.28)	1 (14.29)	1.000	0.559 (0.009–11.81)
	*G*	77 (49.36)	26 (59.09)	–	References	95 (51.08)	8 (57.14)	–	References
	*C*	79 (50.64)	18 (40.91)	0.332	0.675 (0.321–1.399)	91 (48.92)	6 (42.86)	0.875	0.783 (0.215–2.691)
Il-6 *−596G>A* (*rs1800797*)	*GG*	16 (20.51)	7 (31.82)	–	References	21 (22.58)	2 (28.57)	–	References
	*GA*	48 (61.54)	12 (54.55)	0.465	0.571 (0.171–2.036)	56 (60.22)	4 (57.14)	1.000	0.750 (0.099–8.901)
	*AA*	14 (17.95)	3 (13.64)	0.586	1.489 (0.069–2.729)	16 (17.20)	1 (14.29)	1.000	0.656 (0.010–13.80)
	*G*	80 (51.28)	26 (59.09)	–	References	98 (52.69)	8 (57.14)	–	References
	*A*	76 (48.72)	18 (40.91)	0.457	0.729 (0.347–1.510)	88 (47.31)	6 (42.86)	0.969	0.835 (0.229–2.870)
TNF-α *−308G>A* (*rs1800629*)	*GG*	60 (76.92)	17 (77.27)	–	References	72 (77.42)	5 (71.43)	–	References
	*GA*	18 (23.08)	5 (22.73)	1.000	0.980 (0.248–3.304)	21 (22.58)	2 (28.57)	1.000	1.371 (0.122–9.117)
	*AA*	0 (0.00)	0 (0.00)	–	–	0 (0.00)	0 (0.00)	–	–
	*G*	138 (88.46)	39 (88.64)	–	References	165 (88.71)	12 (85.71)	–	References
	*A*	18 (11.54)	5 (11.36)	1.000	0.983 (0.268–2.983)	21 (11.29)	2 (14.29)	0.993	1.310 (0.133–6.525)

Results are expressed as absolute number of patients (percentage). The odds ratio (OR) and 95% confidence intervals (95% CI)

*Il*-*1β* interleukin-1β, *Il*-*6* interleukin 6, *TNF-α* tumor necrosis factor alpha, *Il1 RN* *86 bp* variable number tandem repeat polymorphism of interleukin-1 receptor antagonist, *NEC* necrotizing enterocolitis

**Table 4:** Genotype distribution of the three polymorphisms involved in regulation of arteries tension in infants without and with NEC and without/with NEC surgery-requiring

Gene symbol db SNP	Genotypes/allele	Group without NEC *N* (%)	Group with NEC *N* (%)	*p*	OR 95% CI *p*	Group with NEC without surgery *N* (%)	Group with NEC with surgery *N* (%)	*p*	OR 95% CI *p*
eNOS *894G>T* (*rs1799983*)	*GG*	45 (57.69)	9 (40.91)	–	References	50 (53.76)	4 (57.14)	–	References
	*GT*	31 (39.74)	5 (22.73)	0.964	0.807 (0.193–3.00)	35 (37.63)	1 (14.29)	0.662	0.357 (0.007–3.850)
	*TT*	2 (2.56)	8 (36.36)	0.0004	20 (3.71–208.7)	8 (8.60)	2 (28.57)	0.467	3.125 (0.239–25.81)
	*G*	121 (77.56)	23 (52.27)	–	References	135 (72.58)	9 (64.29)	–	References
	*T*	35 (22.44)	21 (47.73)	0.003	3.157 (1.466–6.726)	51 (27.42)	5 (35.71)	0.696	1.471 (0.368–5.156)
eNOS *−786T>C* (*rs2070744*)	*TT*	32 (41.03)	6 (27.27)	–	References	38 (40.86)	0 (0.00)	–	References
	*TC*	40 (51.28)	11 (50.00)	0.685	1.467 (0.437–5.362)	47 (50.54)	4 (57.14)	–	–
	*CC*	6 (7.69)	5 (22.73)	0.105	4.444 (0.773–24.27)	8 (8.60)	3 (42.86)	–	–
	*T*	104 (66.67)	23 (52.27)	–	References	123 (66.13)	4 (40.00)	–	References
	*C*	52 (33.33)	21 (47.73)	0.118	1.826 (0.871–3.799)	63 (33.87)	10 (60.00)	0.013	4.881 (1.330–21.99)
END-1 *5665G>T* (*rs5370*)	*GG*	52 (66.67)	14 (63.64)	–	References	63 (67.74)	3 (42.86)	–	References
	*GT*	24 (30.77)	7 (31.82)	1.000	1.083 (0.326–3.329)	28 (30.11)	3 (42.86)	0.577	2.250 (0.281–17.70)
	*TT*	2 (2.56)	1 (4.55)	1.000	1.857 (0.029–37.76)	2 (2.15)	1 (14.29)	0.333	10.5 (0.134–243.2)
	*G*	128 (82.05)	35 (79.55)	–	References	154 (82.80)	9 (64.29)	–	References
	*T*	28 (17.95)	9 (20.45)	0.854	1.176 (0.445–2.863)	32 (17.20)	5 (35.71)	0.184	2.674 (0.655–9.549)

Results are expressed as absolute number of patients (percentage). The odds ratio (OR) and 95% confidence intervals (95% CI)

*END-1* endothelin-1, *eNOS* endothelial nitric oxide synthase, *NEC* necrotizing enterocolitis

## Discussion

The aim of our study was to have a homogenous tested probe which is representative for a population of extremely premature infants. The mean gestational age in our population of infants diagnosed with NEC was comparable to the one found in literature (28 + 4 vs. 27 + 3) [26]. In our Department, however, the prevalence of NEC (22%) among newborns under 33 GA was higher than the 6,3% incidence of NEC stage II/III reported by the EPICE research group [27]. These data reflect the statistics for around 300 neonates treated in the Wielkopolska region of Poland where our department is located. US Neonatal Research Network [28] reported a 7% NEC morbidity. According to other American data, NEC prevalence varied from 11 to 30% [11, 26].

The significant variation in data from different NICUs may be related to the disparity in understanding the definition and diagnosing criteria of NEC. Signs and symptoms of the disease tend to be non-specific and involve a high index of suspicion. Recently, this issue has been raised by a group of researchers on the subject [29]. The exclusion of Bell’s stage I—“suspected NEC”—in the analyses has been the proposed [30], similar to the EPICE research. In our study, stage I qualified as a diagnosis of NEC which could have led to the overestimation of exact numbers of NEC cases. High incidence of NEC in our ward might be also a result of statistically significant lower gestational age of patients in Polish NICUs [27] and the fact that our department is of the highest reference in neonatal medicine in our region.

Moreover, in our study, the percentage of surgical NEC (around 30% of NEC cases and 7% of our probe) is higher than in EPICE (2,7% of population) and lower than in the American data (50% VLBW NEC cases) [31]. Again, the 22% mortality rate of our NEC patients is lower than the one generally reported 30% [18]. The mortality in surgical NEC among our patients is lower (28 vs. 45%). These data may reflect the influence of proper decision making concerning timing and the choice of surgical procedures. Rest of the demographic features of our probe seem consistent with the well-documented NEC characteristics and co-morbidities of prematurity, proving that our group was homogenous and representative for the given population.

To date, there have been no studies assessing the association of eNOS: *894G*>*T* and −786 T>C polymorphism genes with NEC. The eNOS gene polymorphism in which guanine (G) is replaced with thymine (T) at nucleotide 894 (exon 7), results in a change of the amino acid sequence Glu298Asp. The −*786T*>*C* polymorphism of the eNOS gene replaces thymine with cytosine in the eNOS gene promoter at position 786. It is thought that in the presence of *894G>T* and −*786T*>*C* polymorphic variants, eNOS enzymatic activity is impaired [32]. Our analysis showed a 20-fold increased prevalence of NEC in infants with the genotype *TT*   (OR 20 (3.71-208.7); *p* = 0.0004) of eNOS *894G*>*T* gene polymorphism. Additionally, there was a higher prevalence of allele *C* carriers of eNOS *786T*>*C* in patients with surgery-requiring NEC. These novel findings suggest that the role of NO in pathogenesis of NEC is significant. Decreased levels of NO in homozygous *TT* of eNOS *894G*>*T* gene polymorphisms and allele *C* carriers of eNOS −*786T*>*C* may predispose to NEC due to ischemia. Franklin et al. analyzed the *6797G*>*A* (rs1800779) eNOS polymorphism. The role of this polymorphism in NEC pathogenesis, however, still remains unclear [33].

Polymorphism *5665G*>*T* of endothelin-1 gene consists of replacing guanine for thymine on position 5665, which affects the amino acid sequence (Lys198Asn). Abnormal production of END-1 in individuals with genotype *GT* and *TT* *5665G*>*T* polymorphism may lead to impaired regulation of vascular smooth muscle tension and ischemia [34]. To our knowledge, there have been no studies assessing the association of NEC and *5665G*>*T* END-1 polymorphism. We could not, however, confirm any role of the END-1 *5665G*>*T* polymorphism in pathogenesis of this disease.

We analyzed the following polymorphisms involved in inflammation pathway: Il-1β *3953C*>*T*, Il-6 −*174G*>*C* and −*596G*>*A*, TNFα −*308G*>A, and *Il-1RN VNTR 86 bp*. Polymorphism Il-1β *3953C*>*T* consists of replacing cytosine with thymine. It leads to appearance of a rarer allele 2 which is connected with higher production of Il-1β [35]. Il-6 −*174G*>*C* is connected with replacing guanine with cytosine at position −174 and Il-6 −*596G*>*A* consists of replacing of guanine with adenosine at position −596. Both homozygotes *CC* of polymorphism Il-6 −*174G*>*C* and AA of polymorphism Il-6 −*596G*>*A* result in decreased production of Il-6 [36]. Polymorphism TNF −*308G*>*A* is caused by replacement of guanine by adenosine, resulting in loss of binding site for transcription factor AP-2 [37]. Treszl et al. found that the prevalence of alleles with guanine–adenine transition in the −308 and −238 positions was the same in NEC and control subjects [38]. Interleukin-1 receptor antagonists competitively inhibit Il-1-induced proinflammatory activity. Its encoding gene is polymorphic, resulting in quantitative differences in both Il-1 receptor antagonist and Il-1β production. The VNTR polymorphism occurs within intron 2 of the human *Il-1RN* gene, consisting of repeats of 86 bp sequence. The number of repeats is of functional significance as these repeats contain binding sites for transcription factors. It was proven that the occurrence of IL1RN*2 is connected with a more severe and prolonged inflammatory response [39]. To our knowledge, the polymorphism of this gene has not been studied in accordance to neonates with NEC. In our analysis, we did not find any significant association.

Our study confirms the finding of Treszl et al. that no significant differences were present in the allelic frequencies of Il-1 and Il-6 genes between NEC and control infants [40]. Large 2009 meta-analysis by Henderson et al. [41] also confirmed that none of the candidate cytokine polymorphisms were significantly associated with NEC. However, Henderson showed that (by the upper limit of the 95% CI of the OR) the common Il-6 −*174G*>*C* had a modest protective effect towards NEC, reducing the odds of developing the disease by half. Previously, this polymorphism has also been linked to an increased Il-6 production in neonatal lymphocytes [42]. Exclusively in neonates, it is thought to alter the promotor region that increases the transcription of Il-6 protein [43]. Decreased susceptibility of invasive infection in preterm infants might also be connected with Il-6 −*174G*>*C* [44] presumably due to a strong immune response to a pathogen. A recent 2015 paper by Franklin et al. showed that 59 Caucasian neonates with Il-6 −*174G*>*C* were over 6 times more likely to have NEC (*p* = 0.013) and over 7 times more likely to have Stage III disease (*p* = 0.011) [33]. This association was not observed in Black neonates. These conflicting data reflect the complexity of immunologic findings in NEC—the observations can suggest rather concomitant than causal role of all the polymorphisms we tested [45].

## Conclusions

Homozygous TT of eNOS *894G*>*T* gene polymorphism are 20 times more likely to develop NEC. In children with surgery-requiring NEC, a higher prevalence of allele C carriers of eNOS *786T*>*C* was found. This study confirms the significant role of NO and ischemia in the pathogenesis of NEC. Identifying gene variants that increase the risk for NEC development may be useful in screening infants with inherent vulnerability and creating strategies for individualized care. New information about NEC pathogenesis may create new opportunities in preventive medicine.

## Electronic supplementary material

Below is the link to the electronic supplementary material.

Supplementary material 1 (XLS 42 kb)
